# Long-Term Effect of Zhenzhu Tiaozhi Capsule (FTZ) on Hyperlipidemia: 2-Year Results from a Retrospective Study Using Electronic Medical Records

**DOI:** 10.1155/2021/6264414

**Published:** 2021-10-18

**Authors:** Xiaoqiang Huang, Huixia Zhan, Jin Yang, Liufen Peng, Shenghua Piao, Lexun Wang, Tian Lan, Xianglu Rong, Jiao Guo

**Affiliations:** ^1^Guangdong Metabolic Diseases Research Center of Integrated Chinese and Western Medicine, Guangzhou 510006, China; ^2^Key Laboratory of Glucolipid Metabolic Disorder, Ministry of Education of China, Guangzhou 510006, China; ^3^Key Unit of Modulating Liver to Treat Hyperlipemia SATCM (State Administration of Traditional Chinese Medicine), Guangzhou 510006, China; ^4^Institute of Chinese Medicine, Guangdong Pharmaceutical University, Guangzhou 510006, China; ^5^Science and Technology Innovation Center, Guangzhou University of Chinese Medicine, Guangzhou 510006, China; ^6^The First Affiliated Hospital of Guangdong Pharmaceutical University, Guangzhou 510080, China; ^7^School of Medical Information and Engineering, Guangdong Pharmaceutical University, Guangzhou 510006, China

## Abstract

The objective of this work was to study the lipid profile (LDL, TC, TG, and HDL) over 2 years in patients with hyperlipidemia in a real-world clinical setting and to describe the dynamical trajectory of lipid profile change in response to lipid-lowering treatment (Zhenzhu Tiaozhi capsule (FTZ) vs. general lipid-lowering treatment, i.e., statins, fibrates, and Xuezhikang). We conducted a retrospective study that included people aged ≥18 years with hyperlipidemia that initiated lipid-lowering treatment between January 2010 and December 2020. Demographic, diagnosis, and laboratory data were retrieved from hospital's electronic medical records, including hospital information system (HIS) and the laboratory information system (LIS). Follow-up trajectories of lipid profile were plotted in a generalized additive mixed model (GAMM) with smooth splines. A total of 839 patients with hyperlipidemia were included. Within 2 years, LDL, TC, and TG descended steadily and gently in the FTZ group (*N* = 99), while the general lipid-lowering treatment (*N* = 740) shortly improved LDL, TC, and TG before 11 weeks and was no longer present around 30 weeks. After 30 weeks, the trajectory of LDL, TC, and TG fluctuated up and down. Also, for HDL, a similar trajectory was observed before 40 weeks between 2 groups, but the FTZ group showed an increasing trend after 40 weeks, while a similar trend was not seen in the general lipid-lowering group. In this study, FTZ was shown to have similar long-term effectiveness as an alternative lipid-lowering treatment to the general lipid-lowering treatment. The findings of this study provide observational evidence for further studies of FTZ, but more prospective studies are needed to determine the impacts of FTZ on lipid profile.

## 1. Introduction

Hyperlipidemia is a common metabolic syndrome worldwide, which is strongly associated with the prevalence of obesity, type 2 diabetes mellitus (T2DM), nonalcoholic fatty liver disease (NAFLD), and hypertension. It is well known that hyperlipidemia, as a cardiovascular risk factor [1], is associated with increased cardiovascular events and death [2]. The management of hyperlipidemia, especially elevated LDL-C, is the vital prevention for patients who have already experienced a cardiovascular event [3], or with cardiovascular risk factors [4]. Although several large studies [3, 5] showed that lipid-lowering treatment had quite good effect in achieving lipid goal setting, several population studies showed that the prevalence of poorly controlled lipid profile remains high [6, 7]. Meanwhile, the visit-to-visit variability in lipid profile has been proven to be a risk factor for cardiovascular diseases [8, 9], which was also not clear among Chinese people.

In China, commonly used lipid-lowering drugs include statins [10], fibrates [11], Xuezhikang (a red yeast rice dietary supplement) [12], and other traditional Chinese medicine (TCM) compounds [13, 14]. Statins are widely used to treat hyperlipidemia and atherosclerosis and are beneficial for primary and secondary prevention of CHD [15]. Fibrates, such as fenofibrate and bezafibrate, are widely used in lipid-lowering therapy for preventing cardiovascular events (CVEs) because of their effect on lowering serum triglycerides [11, 16]. As to Xuezhikang, containing a family of naturally occurring statins, clinical benefits were found in patients with dyslipidemia and CHD in some randomized controlled trials [17, 18]. Meanwhile, it has been recommended in the guideline for China adult dyslipidemia prevention [19]. Although the efficacy of these drugs for the treatment of hyperlipidemia has already been clinically demonstrated in multiple large-scale randomized controlled trials [20], evidence regarding the long-term variability of lipid profile in a real-world clinical setting is still obscure.

Zhenzhu Tiaozhi (FTZ) capsule is a Chinese patent medicine containing eight components of herbs (Supplementary 2: Table S1) [13]. It has been used clinically in treating hyperlipidemia, atherosclerosis, and related diseases for more than 18 years. Previous studies have suggested that FTZ is effective against hyperlipidemia and related diseases [21]. Moreover, *in vitro* and *in vivo* experiments have been shown to regulate glycolipid metabolic disorders (GLMD), decrease serum LDL-C, TC, and TG, and increase HDL-C [13, 22]. However, because of the complex composition of FTZ, it is unknown whether the long-term effects of FTZ on lipids differ from those of general lipid-lowering therapy, and there are no previous studies comparing the long-term effectiveness of TCM compound with general lipid-lowering therapy. Thus, we conducted this retrospective comparative effectiveness research to verify the effects of FTZ and general lipid-lowering therapy based on real-world data from electronic medical records.

## 2. Method

### 2.1. Study Population

**Figure 1:**
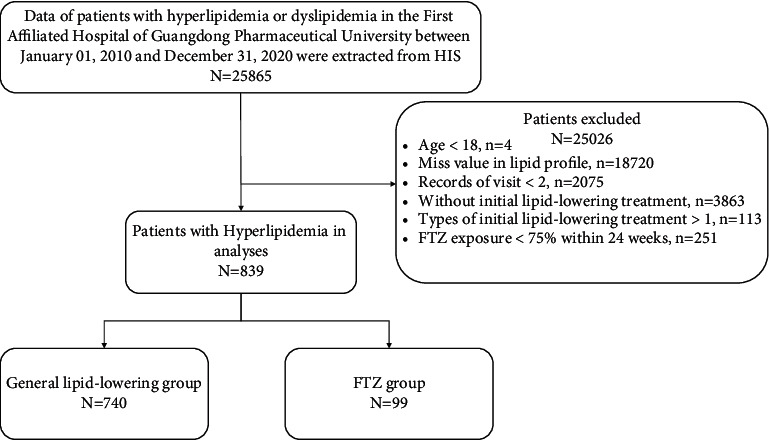
Sample selection flowchart. FTZ exposure <75% in 24 weeks was defined as a prescription dose of FTZ less than 126 days in 24 weeks (168 days).

In this retrospective cohort study, patients who were diagnosed with hyperlipidemia or dyslipidemia (ICD-9-CM codes are shown in Supplementary 2: Table S2) in The First Affiliated Hospital of Guangdong Pharmaceutical University between January 01, 2010, and December 31, 2020, were identified. The First Affiliated Hospital of Guangdong Pharmaceutical University is the first and only hospital with a prescription for FTZ in China. The study baseline was defined as the date hyperlipidemia or dyslipidemia was first diagnosed and lipid-lowering therapy was initiated; follow-up was defined as the subsequent 24 months. Patients with only one visit record or without records of lipid profiles or younger than 18 years at the baseline date were excluded. We also used a drug new-user design [23], so patients without lipid-lowering intervention at the baseline date were also excluded (Figure 1). The study protocol was approved by the first affiliated hospital of Guangdong Pharmaceutical University Ethics Committee.

### 2.2. Data Source

We obtained all demographic, diagnosis, and laboratory data and measurement dates for all study subjects from the hospital information system (HIS) and the laboratory information system (LIS) in the first affiliated hospital of Guangdong Pharmaceutical University. Data extraction was completed in January 2021.

### 2.3. Variable Extraction

Baseline characteristics were collected from HIS, including age, sex, comorbidities, and medication usage. Comorbidities including type 2 diabetes mellitus (T2DM), hypertension, fatty liver, CHD, and arteriosclerosis were collected for analysis based on the recorded ICD-9-CM codes (Supplementary 2: Table S2) from HIS. Data on medication prescriptions for lipid-lowering drugs were extracted from HIS. Laboratory variables including lipid profiles, fasting blood glucose, 2-hour postprandial blood glucose, glycated hemoglobin (HbA1c), serum alanine transaminase (ALT), serum aspartate transaminase (AST), blood urea nitrogen (BUN), and serum creatinine (Cr) were collected from LIS. TC, TG, LDL-C, HDL-C, fasting blood glucose, 2-hour postprandial blood glucose, ALT, AST, BUN, and Cr were measured by using an AU5800 Analyzer (Beckman Coulter Ltd., CA, United States). HbA1c was determined by the HPLC method by utilizing an HbA1c analyzer (Bio-Rad VARIANT II, USA).

### 2.4. Assessment of Drug Exposure

Drug exposure was defined on intention-to-treat (ITT) principles. The general lipid-lowering group was defined by receiving widely used lipid-lowering drugs in China, including statins, fibrates, and Xuezhikang, on the day of diagnosis. The specific names of each class of drugs are shown in the attached table (Supplementary 2: Table S3). The FTZ group received FTZ capsule for at least 24 weeks (days of drug exposure ≥0.75), whose intake methods were four capsules orally and three times daily. The time to initial lipid-lowering treatment was defined as index date. The kind of lipid-lowering drugs and the duration of use of FTZ were also collected.

### 2.5. Statistical Analysis

Baseline lab values were evaluated using records of LIS from three months before to the first day at the index date. Values are presented as the means (standard deviations) or medians (interquartile ranges (IQRs)) for continuous variables, and categorical variables are presented as total numbers and percentages. We first compared the data distribution of each covariate between the general lipid-lowering group and the FTZ groups, using the *t*-test (normal distribution) or Kruskal–Wallis rank-sum test (nonnormal distribution) for continuous variables and *χ*^2^ tests or Fisher's exact test for categorical data.

The dynamical trajectories were analyzed employing generalized additive mixed models (GAMMs) [24] for all lipid profile variables (TC, TG, LDL-C, and HDL), which easily accommodate unbalanced, unequally spaced observations [25], and consequently are ideal tools for analyzing longitudinal data [26]. Covariates included in the models were baseline sex, age, and comorbidity (including T2DM, fatty liver, CHD, hypertension, and arteriosclerosis).

Sensitivity analyses of different lipid-lowering drugs with FTZ were performed. Given that the comparative effectiveness between two groups may differ according to sex, age, and comorbidities, we divided age into three groups and then conducted sensitivity analyses comparing patients among different sex, age, and comorbidities (T2DM, fatty liver, CHD, hypertension, and arteriosclerosis) to evaluate the robustness of our findings. We also conducted sensitivity analyses for other lipid profile variables from LIS (VLDL, ApoAI, ApoB, ApoE, and FFA).

All analyses were performed with *R* (http://www.R-project.org; v3.4.3) and EmpowerStats software (http://www.empowerstats.com; X&Y solutions Inc., Boston, MA) using the package mgcv and nlme. Significance was represented by *P* less than 0.05, two sides.

### 2.6. Ethical Approval

The study was approved by The Institutional Ethics Committee (ICE) of The First Affiliated Hospital of Guangdong Pharmaceutical University, China (ICE approval ID: 2021-ICE-8). The procedures were performed in accordance with the Declaration of Helsinki and relevant guidelines and regulations. This retrospective study was conducted after all patients had completed the visit, and the data were anonymous. Therefore, informed consent was not required and was specifically waived by the ICE.

## 3. Result

### 3.1. Baseline Characteristics

Electronic medical records from January 1, 2010, to December 31, 2020, were screened. Of 839 patients who received one lipid-lowering treatment, 99 patients initiated FTZ as monotherapy and 740 patients initiated general lipid-lowering drug, such as statins (*N* = 572), fibrates (*N* = 114), or Xuezhikang (*N* = 54); each patients had multiple lab tests. Baseline characteristics for study participants at first index date are given in Table 1. The mean sample age was 61.4 ± 14.15 years, and 57.58% were male in the FTZ group. The mean sample age was 64.1 ± 13.61 years, and 50.54% were female in the general lipid-lowering group.

At index date, 38.65%, 29.19%, 33.51%, 64.46%, and 55.95% of patients in the general lipid-lowering group had diagnoses of T2DM, fatty liver, CHD, hypertension, and arteriosclerosis, respectively. 48.48%, 37.37%, 61.62%, 72.73%, and 60.61% of patients in the FTZ group had diagnoses of T2DM, fatty liver, CHD, hypertension, and arteriosclerosis, respectively.

### 3.2. Longitudinal Trends in Lipid Profile between the FTZ Group and the General Lipid-Lowering Group

**Figure 2:**
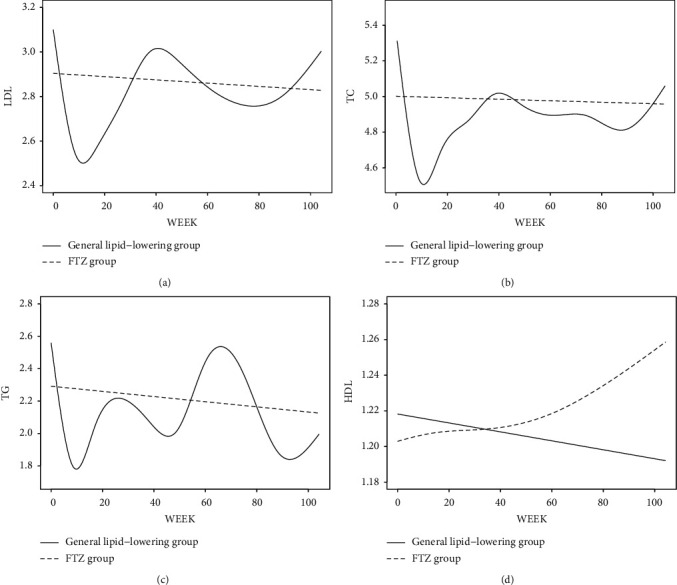
Lipid profile trajectories two years after the initial treatment (adjusted for sex, age, and comorbidity).

Figure 2 shows a continuous temporal trajectory of lipid profile for each group using all available lab test values within a date range from index date to 2 years later. The observed short-term improvement in LDL, TC, and TG for the general lipid-lowering group to the FTZ group peaks at 11 weeks (in vision) and was no longer present around 30 weeks (in vision). After 30 weeks, the trajectory of LDL, TC, and TG fluctuates up and down, while LDL, TC, and TG descended steadily and gently in the FTZ group. Also, for HDL, a similar trajectory was observed before 40 weeks between 2 groups, but the FTZ group showed an increasing trend after 40 weeks. Overall, lipid profile (except HDL) changes occurred early and rapidly from baseline in the general lipid-lowering treatment group, and the difference in trajectory between the two groups disappeared after 30 weeks. Meanwhile, the long-term effect of FTZ on HDL showed a different trend from that of the general lipid-lowering group.

### 3.3. Sensitivity Analyses

Because different types of lipid-lowering drugs may have different effects on different components of lipid profiles, we performed a subgroup analysis of the four lipid-lowering drugs included in this study. From the results of the subgroup analysis (Supplementary 2: Figure S1), we found that the four lipid-lowering drugs showed different curve characteristics. Statins remain the most effective drugs for LDL-C lowering before 11 weeks but show a similar treatment effect to the other three drugs after 30 weeks. Fibrates have a superior improvement for TG before 11 weeks, but this effect does not last until the end of 2 years. Statins and fibrates exert similar effects on TC better than FTZ and Xuezhikang, but the trajectory tended to be similar among 4 groups after 30 weeks. Close to the overall comparison, the FTZ group showed an increasing trend of HDL, but similar changing trends were not seen in the other 3 groups.

Because of the association between age and blood lipid levels [27], we divided FTZ groups' individuals into three groups (youth, middle-aged people, and elderly people), and the above models were applied in these three groups, respectively (Supplementary 2: Figure S2). The trajectories of LDL and HDL in youth and middle-aged FTZ users showed opposite U-shaped features. The trajectory of TC in young people is the same as that of LDL, showing a decline followed by an increase, with the break point at 40 weeks (in vision). The trajectory of TC in middle-aged people showed a decline followed by an increase, while the youth and elderly showed a continuous decline. Sex and comorbidities did not change the main outcome of comparative effectiveness between the FTZ group and the general lipid-lowering group (Supplementary 2: Figures S3–S8). The trajectory of VLDL and ApoB showed similar characteristics to TC, while ApoAI was similar to HDL. The trajectory of ApoE in FTZ users showed a decline and FFA increased slightly, while the general lipid-lowering group showed U-shaped features in ApoE and L-shaped in FFA (Supplementary 2: Figure S9).

## 4. Discussion

Our study identified 839 patients who received one kind of lipid-lowering drug, and 2 years' lipid profile records were tracked. The level of lipid control appears to be approximate over the long-term follow-up, with differences occurring only because of the different initial drugs. Our study revealed that FTZ might have a mild lipid-lowering effect in patients with hyperlipidemia and no excessive fluctuations in blood lipid levels, compared with general lipid-lowering drugs. For other lipid profile variables, the trajectories of VLDL, ApoAI, and ApoB were consistent with their major components, but ApoE and FFA have opposite trajectories in the FTZ group. Because the composition of lipids and FTZ is quite complex, there may be different mechanisms of action for different components of the lipid profile. In the sensitivity analyses of different drugs, FTZ exhibited similar curve characteristics to Xuezhikang, and these two drugs' curves exhibited consistent characteristics with the primary results compared to statins and fibrates. Sex and comorbidities did not change the main outcome of comparative effectiveness between the FTZ group and the general lipid-lowering group, but the trajectories of FTZ group were better in male and individuals without comorbidities.

Lipid-lowering therapy for hyperlipidemia and the prevention of heart attacks and strokes has been widely proved, when used appropriately and persistently [5]. We identified three types of generally used lipid-lowering drugs, including statins, fibrates, and Xuezhikang, through the HIS system and compared the effectiveness between them and FTZ. It has been proven that all these drugs could reduce the incidence of cardiovascular events and lower blood lipid levels [28]. The cardiovascular benefits of statins have been demonstrated in observational studies. Based on the 20% threshold for cardiovascular risk, for women with any statin to prevent one case of cardiovascular disease over five years, the value was 37 (95% confidence interval: 27–64), and for men, the respective value was 33 (95% CI: 24–57) [29]. Fibrate therapy can reduce TG levels by as much as 50 percent or more [30]. A meta-analysis of 10 randomized trials (36,489 patients; both primary and secondary prevention trials) found that the long-term use of fibrates significantly reduces the occurrence of nonfatal MI by about 22% (*P* < 0.00001) [31]. Xuezhikang was also commonly prescribed for hyperlipidemia other than statins and fibrates in China. Several studies have shown that Xuezhikang resulted in decreases in LDL-C (∼27% reduction) after 12 weeks treatment [18] and significantly decreased cardiovascular events (30%) and total mortality (33%) in patients who have had a previous myocardial infarction compared with placebo on an average of 4.5 years [17]. However, more lipid-lowering drugs' effectiveness in the real world remains unclear. As with our studies using HIS and LIS, it is essential and valuable to apply real-world data to conduct comparative efficacy studies of herbal medicines which are already clinically used compared with common lipid-lowering drugs.

Herbal medicine is a valuable source of novel chemical structures and contains multiple active ingredients that have the possibility of being beneficial for disease prevention and treatment. Based on traditional Chinese medicine concept, our group proposed “Tiao Gan Qi Shu Hua Zhuo” (modulating Gan, trigging key metabolic system to resolve pathogenic factors such as phlegm retention and dampness) in prevention and control of hyperlipidemia [32]. Also, the FTZ was innovated under these rationales. Among the eight herbs in prescription FTZ, there were sixteen constituents that showed potential effects towards the hyperlipidemia-related targets, and Rhizome Coptidis plays the most important role in the whole effect towards hyperlipidemia [13]. FTZ realizes the regulation of the target, hepatic HMGCR, cholesterol 7-alpha hydroxylase (CYP7A1), hepatic lipase, low-density lipid receptor by regulating the expression of PPAR*α*, liver *X* receptor alpha, and other nuclear receptors, resulting in the adjustment of lipid absorption, synthesis, transformation, transport, decomposition, and excretion of metabolic processes [33]. The identification of responsible compounds for each pharmacological activity of herbal medicine is tough because of the complexity of synergistic, additive, or antagonistic pharmacological effects of many constituents in herbal products, whereas synthetic agents are not. Precisely for this reason, FTZ exhibits lipid-lowering effects by regulating various targets [34], compared with synthetic agents, and shows unique characteristics of lipid-lowering effects, which are also reflected in the trajectories of lipid profiles in our study.

The absolute benefits of lipid-lowering therapy depend on the absolute reduction in LDL-C that is achieved. However, several cross-sectional studies have demonstrated that many patients with hyperlipidemia have not achieved their optimal management goals and have difficulty following recommendations for self-management [6, 35]. This perspective seems to explain the finding in the present study that the trajectory of the lipid profile in the general lipid-lowering group was characterized by a rapid decrease, followed by an increase, and then an up-and-down fluctuation. Compared with prospective studies, retrospective studies from real-world data may avoid the Hawthorne effect [36], which may prove that the dynamical trajectory of lipid profile change in both lipid-lowering treatment groups was real.

Lipid-lowering therapy has been shown to reduce vascular disease risk as it continues to be taken, and these benefits persist long term because of controlling LDL-C at an appropriate level [5]. Large randomized clinical trials have documented its benefits in patients at risk for or presenting established cardiovascular disease (CVD) as first-line lipid-lowering therapy. When these conclusions are based on the results of well-designed large-scale randomized controlled trials, the value of retrospective observational studies for the assessment of the effectiveness of treatment is more limited. However, it is still necessary to collect real-world data to compare the effectiveness of new therapies with existing ones, and in the meantime, the trajectory of lipid profile when receiving lipid-lowering treatment is still unclear in the real world.

Our study has some strengths. First, in this single-center study, for the first time, we used electronic medical records to depict trends in lipid changes in hyperlipidemia patients who had repeat visits. Also, we found that although statins and fibrates appear to be very rapid in lowering lipids in the short term, the long-term effects are similar to those of FTZ and Xuezhikang. Second, the trajectory of the general lipid-lowering treatment group shows a rapid decline followed by a rebound, which suggests possible poor adherence to medical therapies or a lack of management of lipid-lowering goals. Third, the results obtained from this study provide evidence from an observational retrospective study to compare the effects of FTZ with general lipid-lowering therapy, when large-scale evidence from randomized controlled trials does not exist. Fourth, the lipid profile and its components (TC, LDL-C, HDL-C, and TG) are some of the most commonly ordered laboratory tests in clinical practice for dyslipidemia and cardiovascular disease patients. Our study suggests that extraction of previous lipid profile data from hospital's LIS could be a possible way of monitoring the effectiveness of lipid-lowering drugs. Meanwhile, this research approach may also be a possible way to provide real-world evidence for new drug research or re-evaluation of marketed drugs.

However, there were several limitations to the present study. First, the lipid profile was collected through passive surveillance of electronic records rather than dedicated study measurements. The methods of outcome collection resulted in missing data that could influence results. In this regard, prospective studies are necessary to reproduce the results that are generated in this study. Second, even if we adjusted sex, age, and comorbidity to show the smooth splines are stable and used drug new-user design to avoid unmeasured drug effect, some unforeseen confounders (e.g., prehospital medication and alcohol consumption), because this study was conducted in a single center, may still potentially alter the lipid-lowering treatment effects. In such circumstances, what we found may well yield associations of treatment with health outcomes, but that is not causal because of the potential biases that are inherent in observational studies. Third, the data from this study did not include cardiovascular events due to limitations of HIS, but prospective studies could be conducted based on this study to verify the possible cardiovascular benefit of FTZ.

## 5. Conclusion

In this real-world study, FTZ as an optional lipid-lowering drug resulted in similar effectiveness of improving lipid profile among hyperlipidemia patients in the long-term treatment compared with general lipid-lowering treatment. What we found in the present study gives observational evidence for further study of FTZ, and the effects of this drug still need more prospective studies to prove.

## Figures and Tables

**Table 1:** Characteristics of patients in statin and nonstatin groups on index date.

Parameters	General lipid-lowering group (*N* = 740)	FTZ group (*N* = 99)	*P* value^a^
Clinical characteristics			
Age, years (mean (SD))	64.1 (13.61)	61.4 (14.15)	0.019
Male gender, *n* (%)	374 (50.54%)	57 (57.58%)	0.188

Laboratory examination			
FBS, mmol/L (mean (SD))	5.99 (2.25)	6.17 (2.81)	0.570
2hPG, mmol/L (median (IQR))	8.62 (6.57–12.49)	7.70 (6.64–9.77)	0.378
HbA1c, % (mean (SD))	6.57 (1.75)	6.40 (1.82)	0.507
TC, mmol/L (mean (SD))	5.38 (1.43)	5.12 (1.57)	0.166
TG, mmol/L (median (IQR))	1.94 (1.30–2.92)	1.50 (0.99–2.29)	0.002
LDL, mmol/L (median (IQR))	3.00 (2.26–3.97)	2.84 (2.20–3.51)	0.398
HDL, mmol/L (mean (SD))	1.20 (0.32)	1.29 (0.39)	0.159
ALT, mmol/L (median (IQR))	18.00 (14.00–27.00)	21.00 (14.15–26.50)	0.862
AST, mmol/L (median (IQR))	21.00 (16.00–27.00)	20.00 (17.00–24.00)	0.398
Cr, *μ*mol/L (median (IQR))	75.00 (62.00–88.00)	65.50 (57.00–83.00)	0.020
BUN, mmol/L (median (IQR))	5.49 (4.54–6.75)	5.14 (4.40–5.84)	0.114

Comorbidities			
T2DM, *n* (%)	286 (38.65%)	48 (48.48%)	0.060
Fatty liver, *n* (%)	216 (29.19%)	37 (37.37%)	0.096
CHD, *n* (%)	248 (33.51%)	61 (61.62%)	<0.001
Hypertension, *n* (%)	477 (64.46%)	72 (72.73%)	0.104
AS, *n* (%)	414 (55.95%)	60 (60.61%)	0.380
Lipid-lowering treatments			
Statins	572 (77.30%)	—	—
Fibrates	114 (15.40%)	—	—
Xuezhikang	54 (7.30%)	—	—
FTZ	—	99 (100%)	—

FBS, fasting blood sugar; 2hPG, 2-hour postprandial blood glucose; HbA1c, hemoglobin A1c; TC, total cholesterol; TG, total triglyceride; LDL, low-density lipoprotein; HDL, high-density lipoprotein; ALT, alanine transaminase; AST, aspartate transaminase; Cr, creatinine; BUN, blood urea nitrogen; T2DM, type 2 diabetes mellitus; CHD, coronary heart disease; AS, arteriosclerosis; FTZ, Zhenzhu Tiaozhi capsule; IQR, interquartile range. ^a^*P* values were calculated by the *t*-test (normal distribution) or Kruskal–Wallis rank-sum test (nonnormal distribution) for continuous variables and *χ*^2^ tests or Fisher's exact test for categorical data.

## Data Availability

The raw/processed data required to reproduce these findings cannot be shared at this time as the data also form part of an ongoing study.

## Abbreviations

ALT: Serum alanine transaminase
ApoAI: Apolipoprotein A-I
ApoE: Apolipoprotein E
AST: Serum aspartate transaminase
BUN: Blood urea nitrogen
CHD: Coronary heart disease
Cr: Serum creatinine
CVD: Cardiovascular disease
CVEs: Cardiovascular events
FFA: Free fatty acid
FTZ: Zhenzhu Tiaozhi capsule
GAMMs: Generalized additive mixed models
GLMD: Glycolipid metabolic disorders
HIS: Hospital information system
ICE: The Institutional Ethics Committee
ITT: Intention-to-treat
LIS: Laboratory information system
NAFLD: Nonalcoholic fatty liver disease
T2DM: Type 2 diabetes mellitus
TCM: Traditional Chinese medicine
VLDL: Very-low-density lipoprotein cholesterol.

## Supplementary Materials

Supplementary Materials Supplementary 1: a list of STROBE checklist for cohort. Supplementary 2: components of FTZ, ICD codes, trade names of drugs, and figures of sensitivity analyses.
