# Supplementary material for: Long-Term Effect of Zhenzhu Tiaozhi Capsule (FTZ) on Hyperlipidemia: 2-Year Results from a Retrospective Study Using Electronic Medical Records
Source: Evid Based Complement Alternat Med. 2021 Oct 18;2021:6264414. doi: 10.1155/2021/6264414 (PMC8545570; doi:10.1155/2021/6264414)
Supplement: Supplementary Materials — Supplementary 1: a list of STROBE checklist for cohort. Supplementary 2: components of FTZ, ICD codes, trade names of drugs, and figures of sensitivity analyses. [file 6264414.f1.zip › 6264414.f1/supple 2.docx]

# supple 2

| Table S1 The components of FTZ in Chinese, and Latin names | |
| --- | --- |
| Chinese Pin Yin name | Latin binomial name |
| Nv zhen zi | Fructus Ligustri Lucidi |
| Huang lian | Rhizoma Coptidis |
| Dan shen | Radix Salviae Miltiorrhizae |
| San qi | Radix Notoginseng |
| Bai zhu | Rhizoma Atractylodis Macrocephalae |
| Fo shou | Fructus Citri Sarcodactylis |
| Da ji | Radix Cirsii Japonici |
| Du zhong | Cortex Eucommiae |

| Table S2 ICD-9-CM codes used to classify comorbidities | |
| --- | --- |
| Comorbidities | ICD-9-CM codes |
| Hyperlipidemia | E78.000, E78.200, E78.208, E78.400, E78.500, E78.5, E78.501 |
| T2DM | E11.200, E11.201+N08.3*, E11.300, E11.301+H36.0*, E11.302+H28.0*, E11.303+H22.1*, E11.400, E11.401+G63.2*, E11.402+G99.0*, E11.403+G63.2*, E11.404+G99.0*, E11.405+G73.0*, E11.406+G99.0*, E11.500, E11.501+I79.2*, E11.502+I79.2*, E11.503, E11.504, E11.505, E11.600, E11.601+M14.2*, E11.602+M14.6*, E11.603+L99.8*, E11.700, E11.800, E11.900, E14.200, E14.300, E14.400, E14.500, E14.600, E14.700, E14.800, E14.900 |
| FattyLiver | K76, K76.000, K76.001 |
| Coronary heart disease | I25.000, I25.100, I25.101, I25.102, I25.103, I25.104, I25.200, I25.201, I25.202, I25.203, I25.204, I25.205, I25.206, I25.207, I25.208, I25.800, I25.801, I25.900, I25.901, I25.902, Z95.501, Z95.101 |
| Hypertension | I10.x00, I10.x01, I10.x03, I10.x04, I10.x05, I10.x07, I10.x08, I10.x09, I10.x11, I11, I12.000, I12.900, I13, I67.400, H35.004 |
| Arteriosclerosis | I67.200, I67.201, I67.202, I67.203, I70.000, I70.002, I70.100, I70.200, I70.201, I70.202, I70.203, I70.204, I70.205, I70.207, I70.208, I70.209, I70.800, I70.802, I70.803, I70.804, I70.805, I70.806, I70.900 |

ICD-9-CM was the diagnostic coding available in the first affiliated hospital/school of clinical medicine of Guangdong Pharmaceutical University during the period of this study.

| Table S3 Extraction of lipid-lowering drugs form HIS | |
| --- | --- |
| Statins | simvastatin, rosuvastatin, atorvastatin, pravastatin, fluvastatin |
| Fibrates | fenofibrate, bezafibrate |


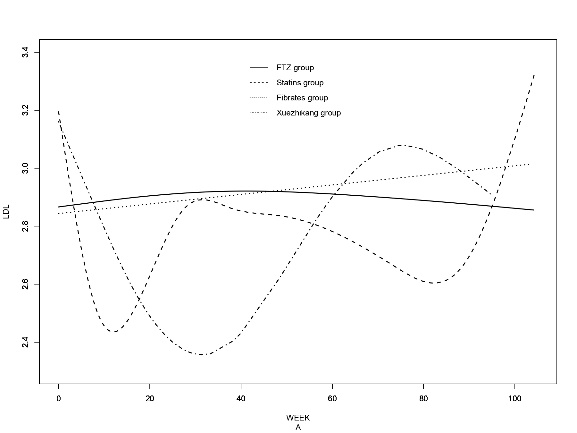

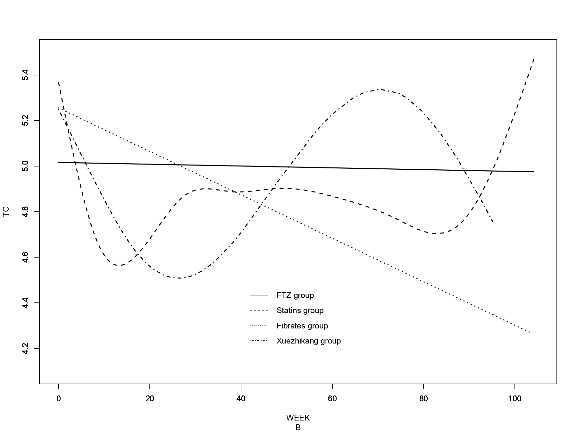

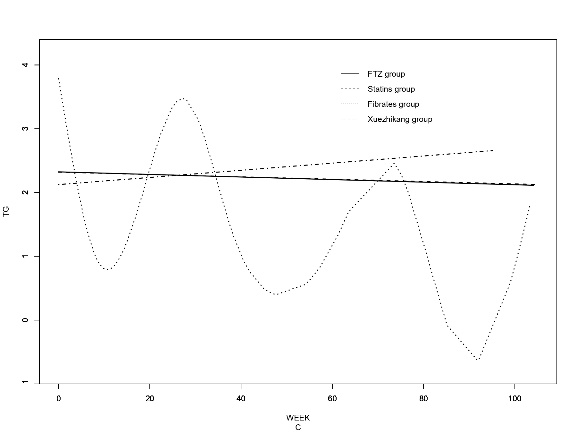

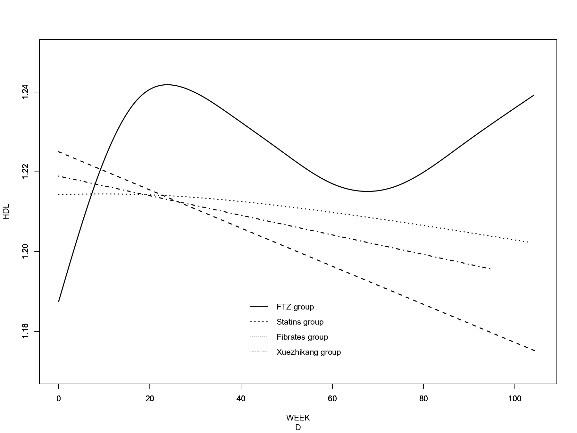


Figure S1. Lipid profile trajectories two years after the initial treatment divided by Statins, Fibrates, Xuezhikang and FTZ. Adjusted for age and comorbidities.


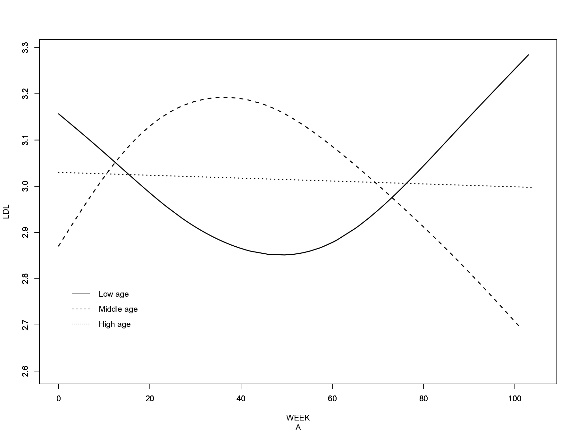

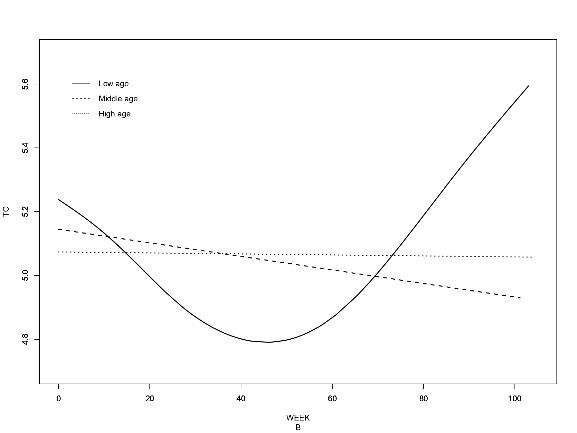

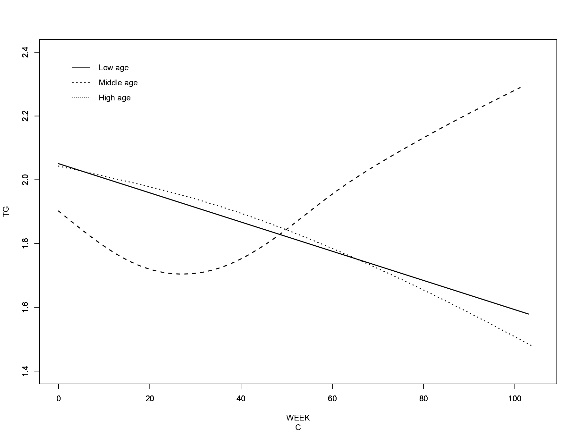

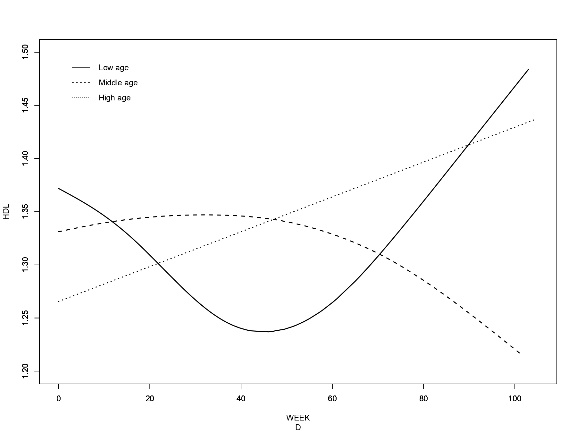


Figure S2. Lipid profile trajectories two years after the initial FTZ treatment divided by age. Adjusted for sex and comorbidities.


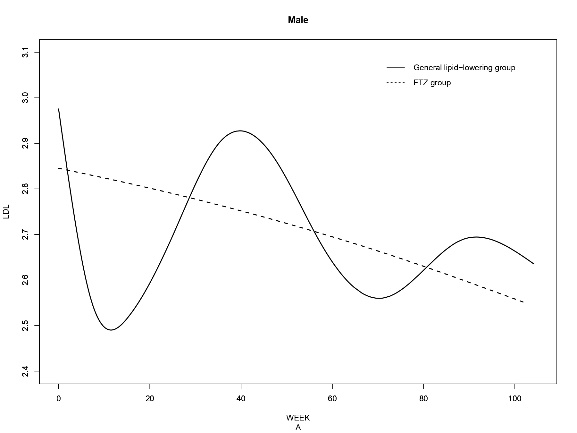

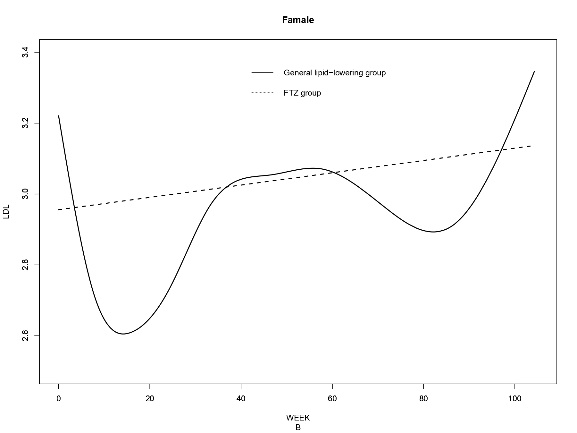

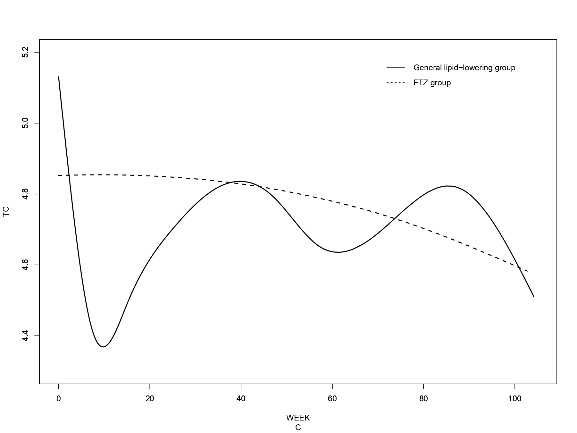

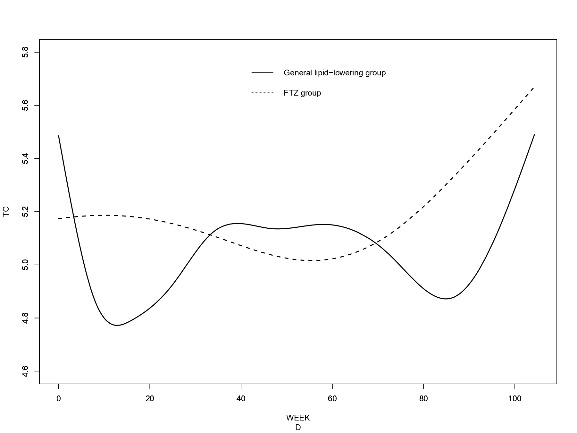

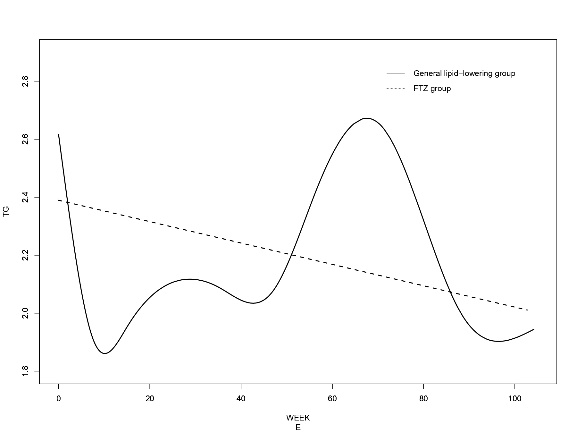

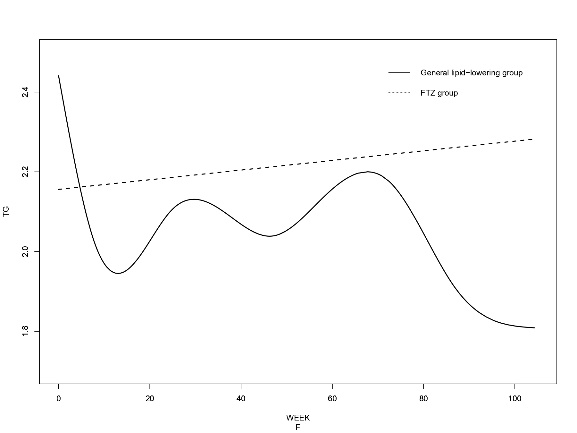

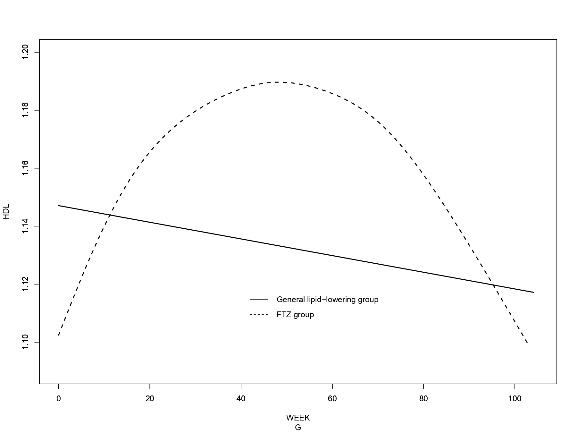

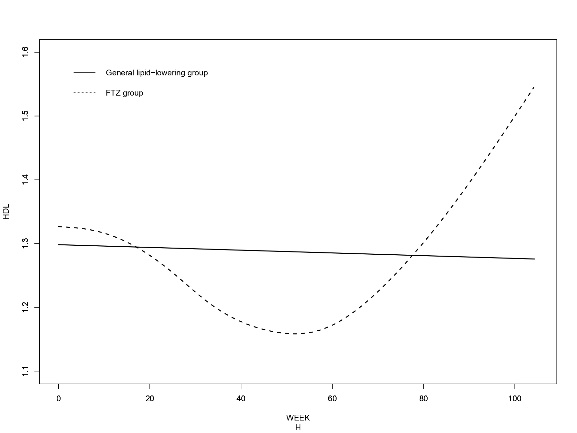


Figure S3. Lipid profile trajectories two years after the initial treatment divided by sex. Adjusted for age and comorbidities.


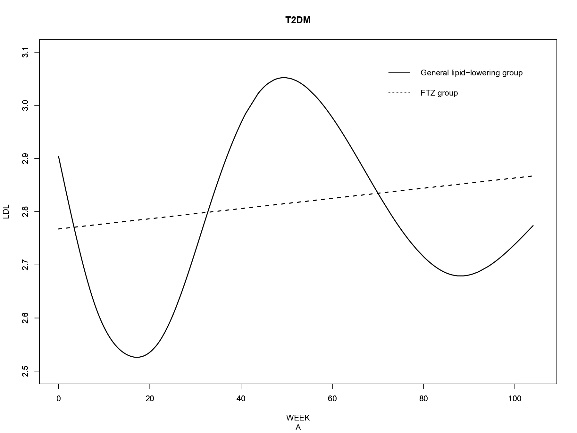

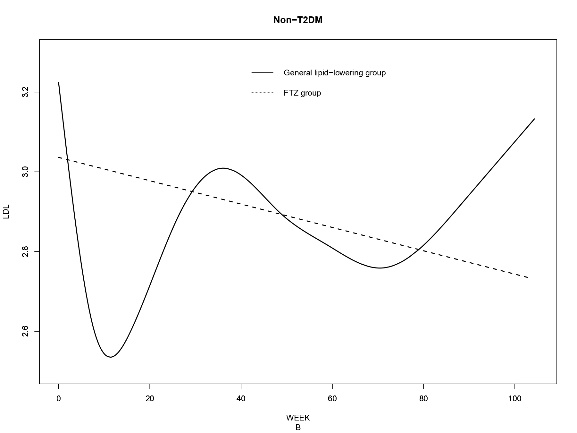

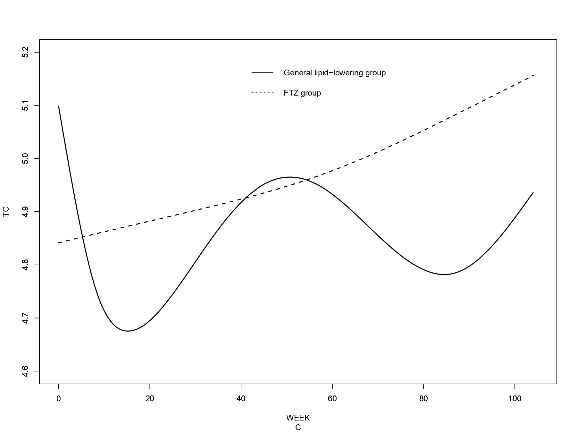

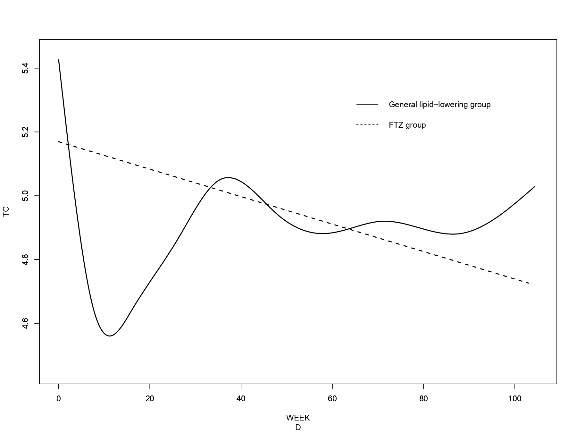

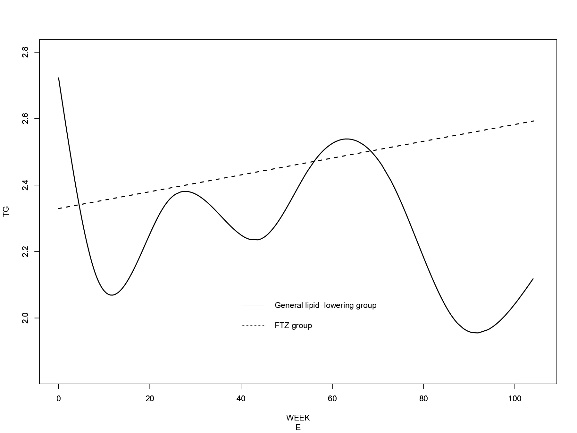

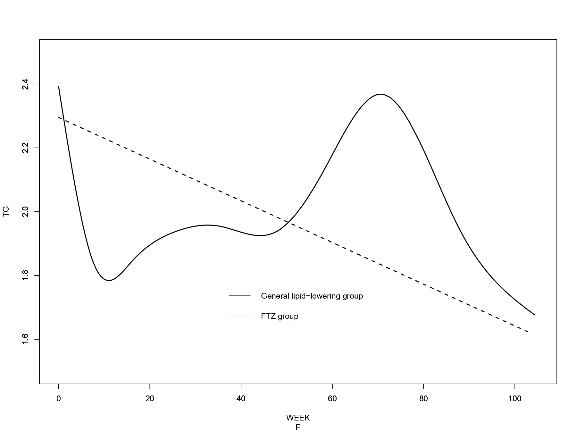

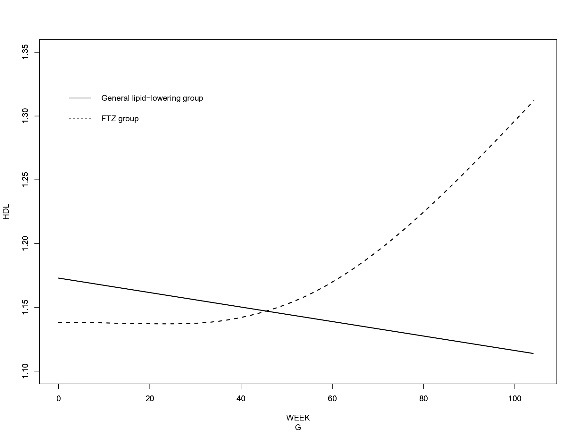

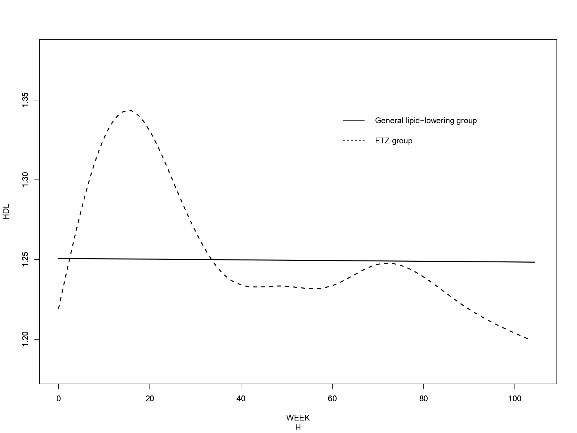


Figure S4. Lipid profile trajectories two years after the initial treatment divided by T2DM. Adjusted for age, sex and comorbidities.


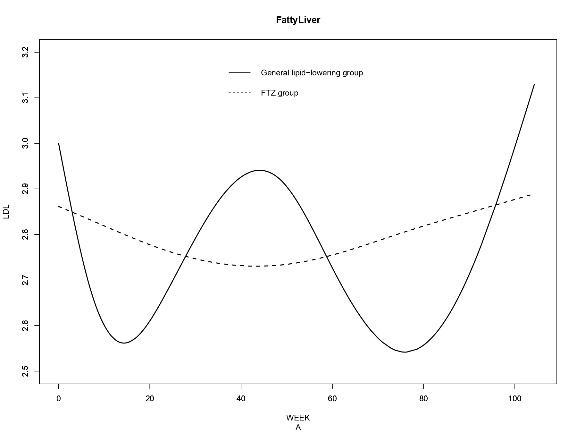

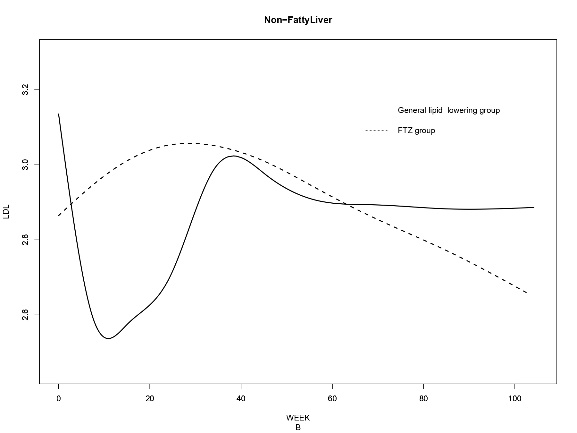

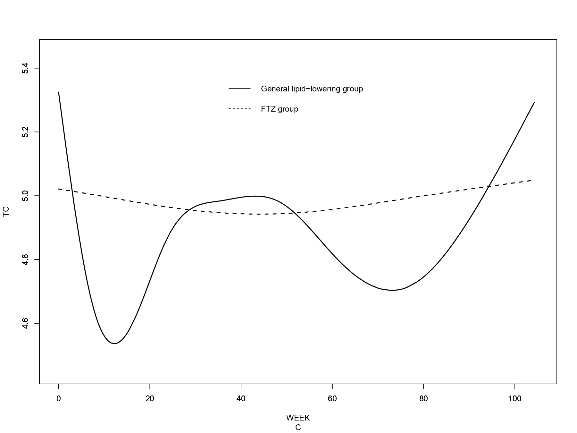

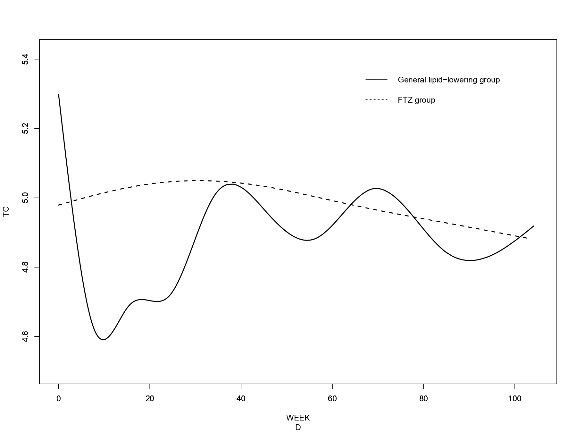

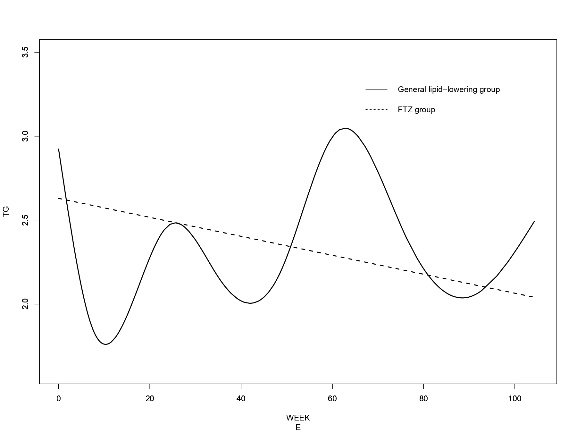

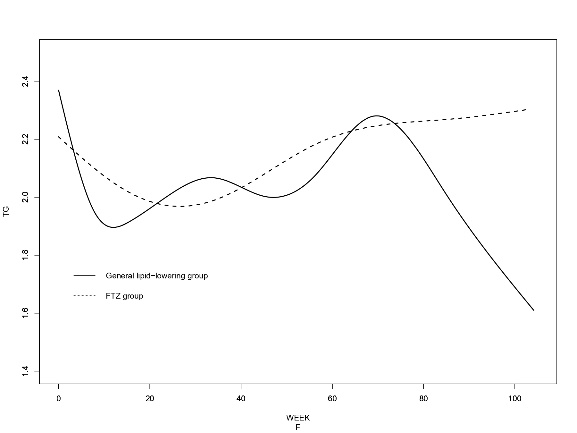

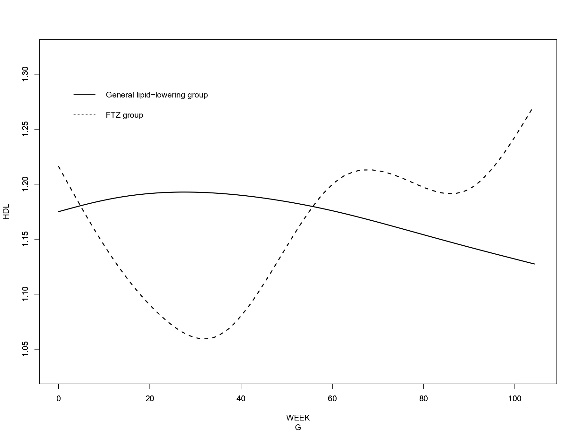

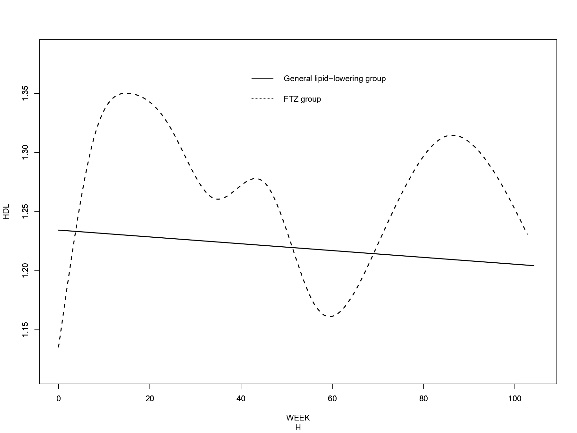


Figure S5. Lipid profile trajectories two years after the initial treatment divided by FattyLiver. Adjusted for age, sex and comorbidities.


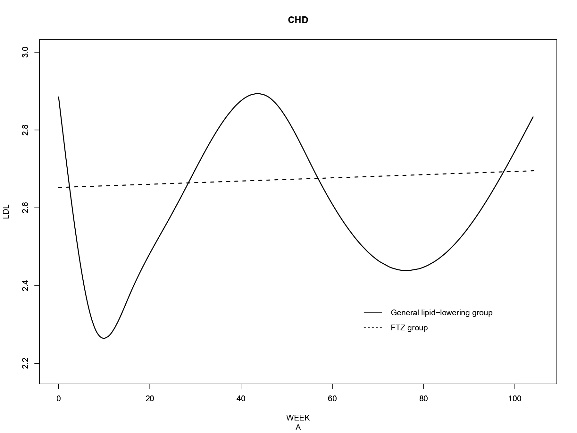

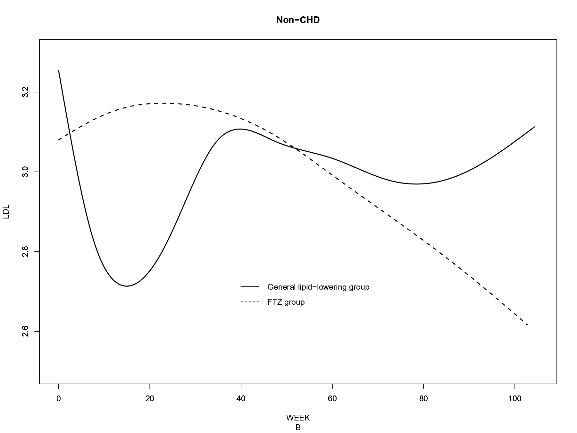

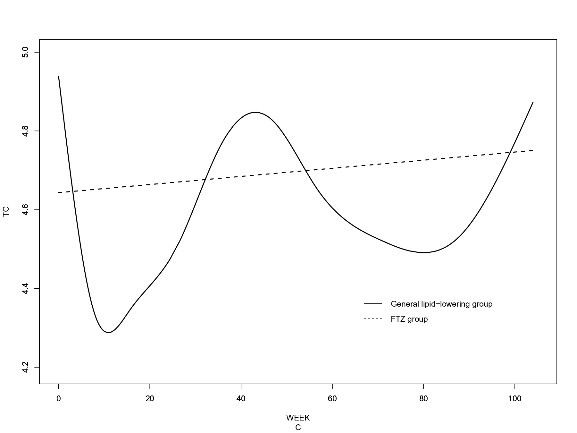

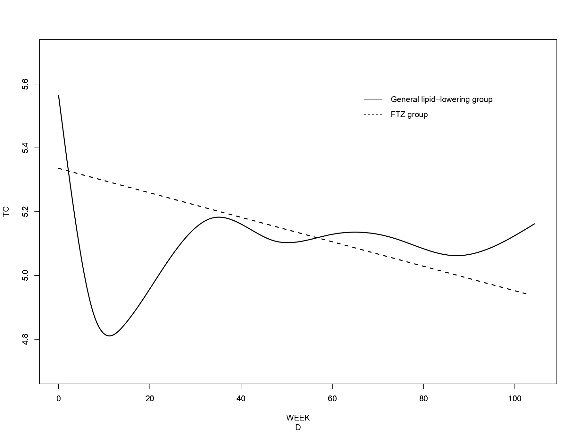

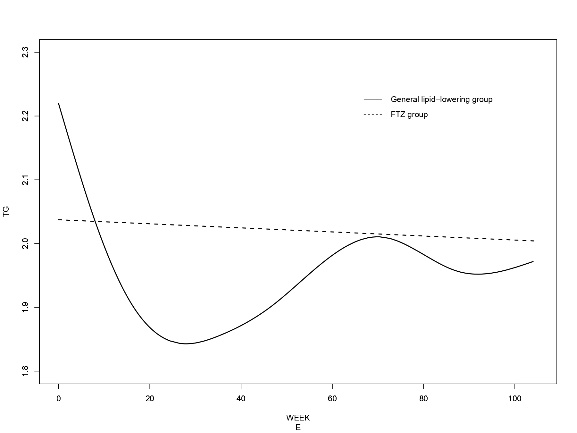

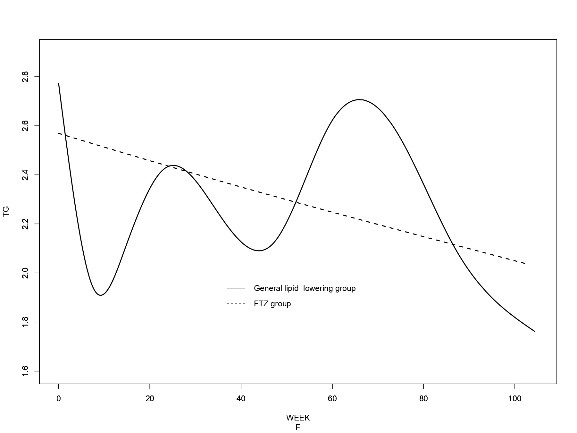

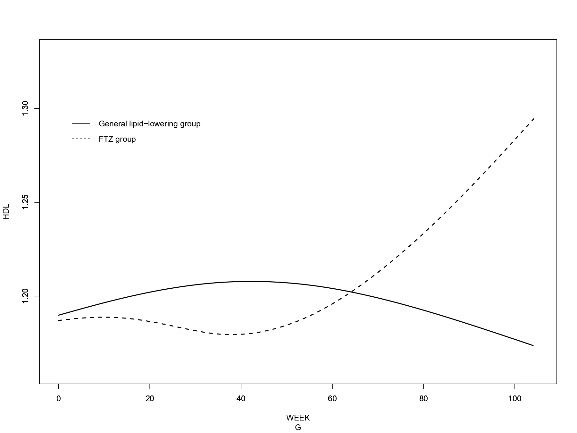

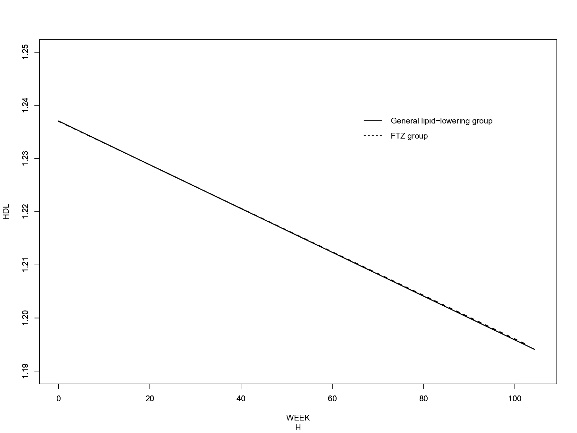


Figure S6. Lipid profile trajectories two years after the initial treatment divided by CHD. Adjusted for age, sex and comorbidities.


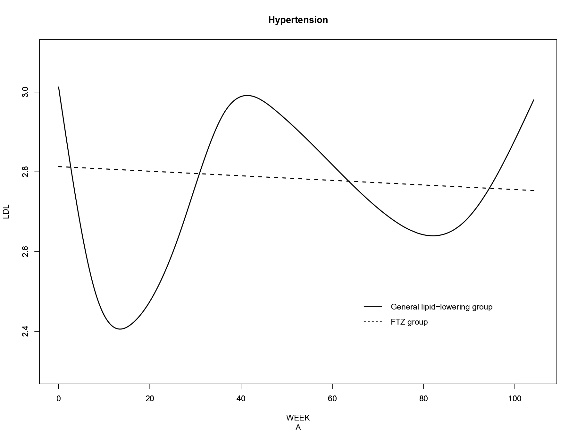

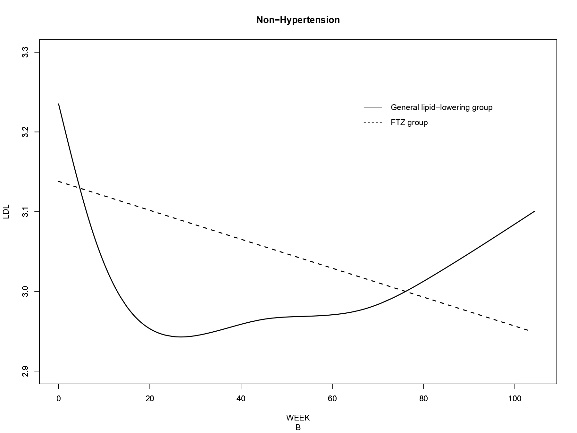

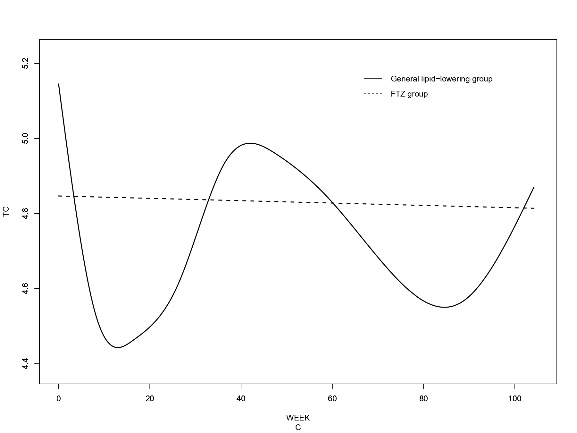

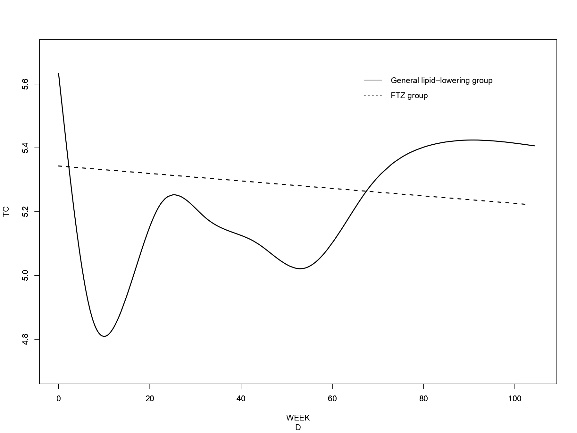

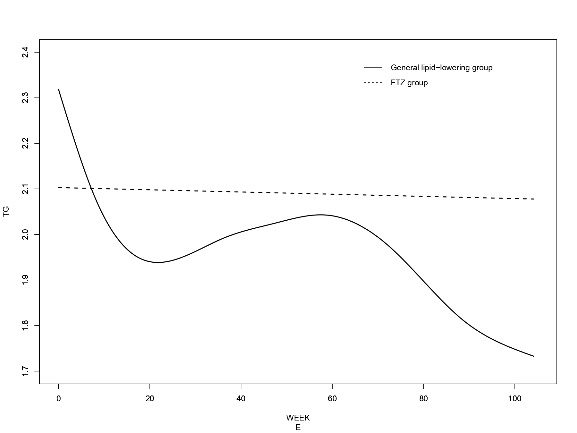

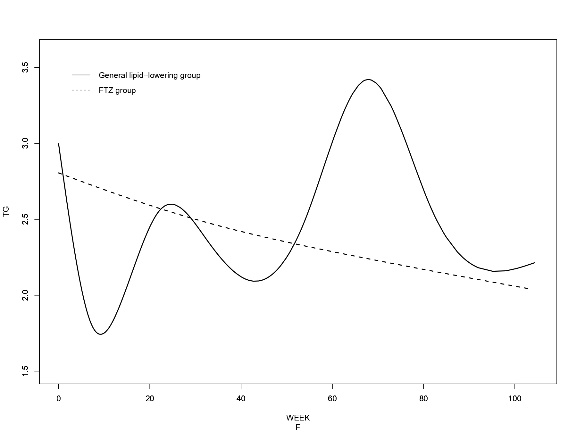

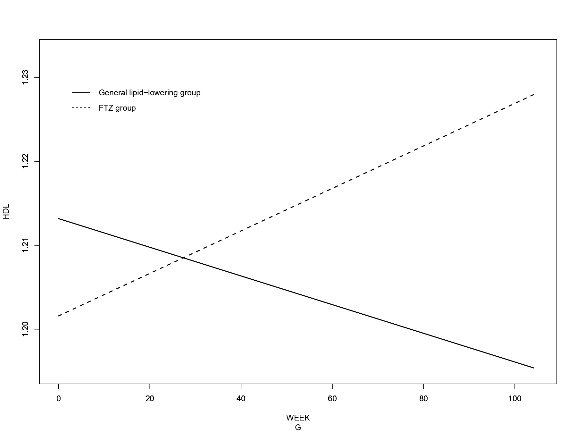

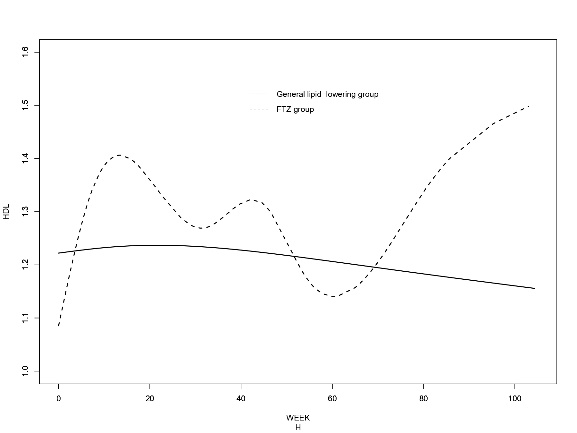


Figure S7. Lipid profile trajectories two years after the initial treatment divided by Hypertension. Adjusted for age, sex and comorbidities.


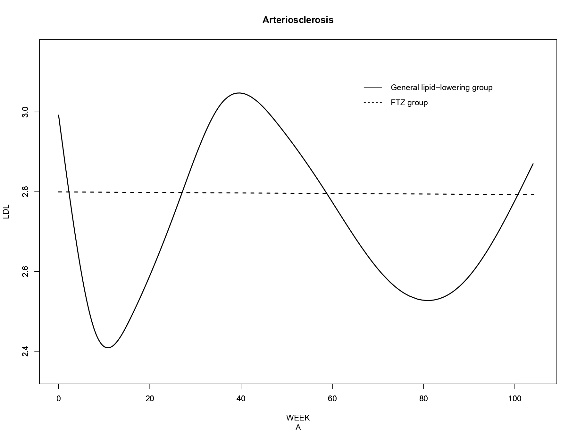

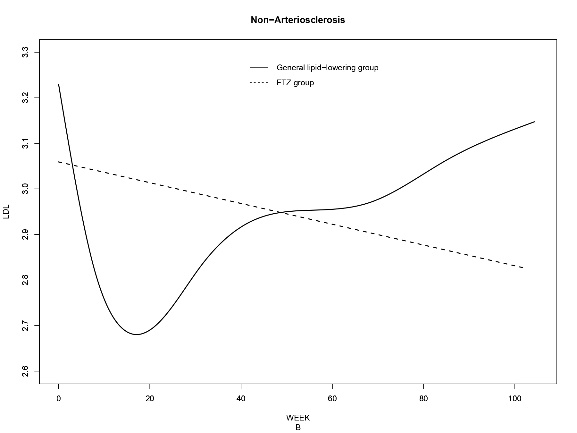

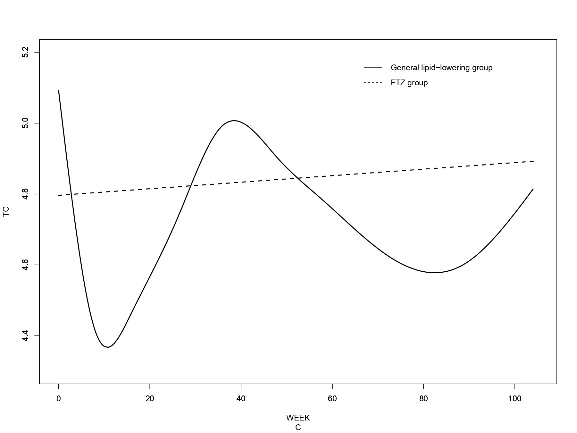

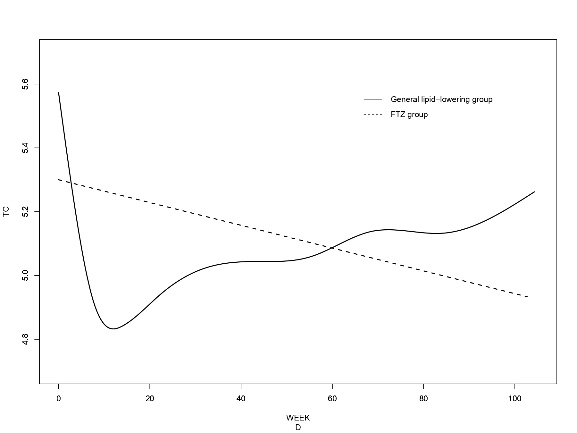

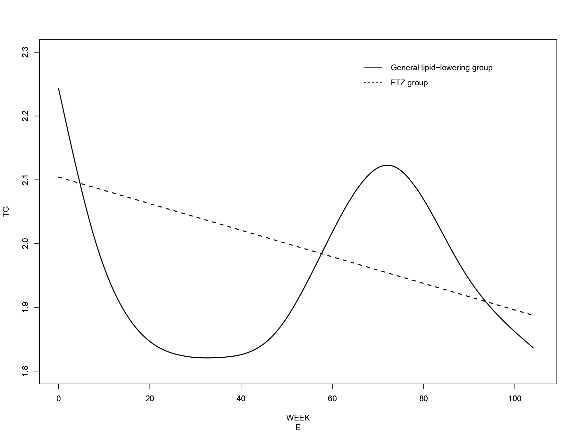

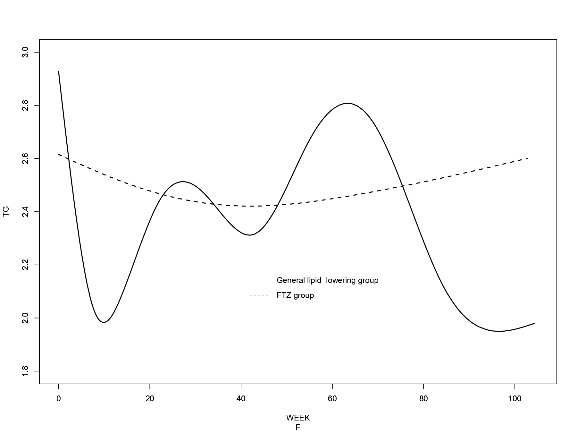

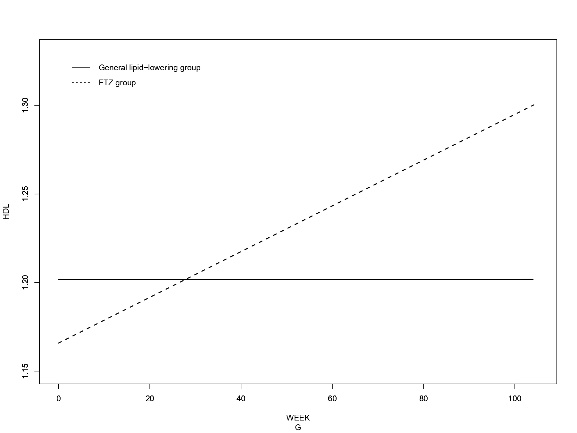

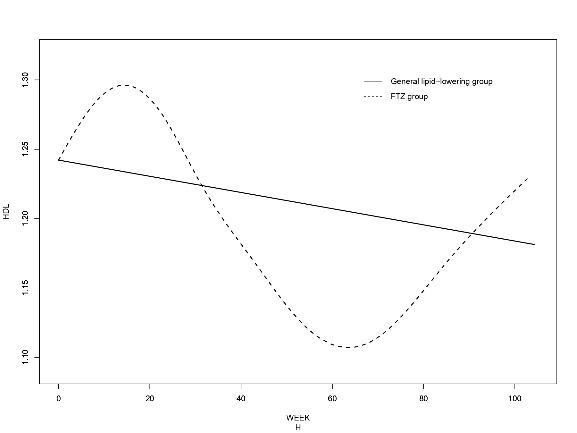


Figure S8. Lipid profile trajectories two years after the initial treatment divided by Arteriosclerosis. Adjusted for age, sex and comorbidities.


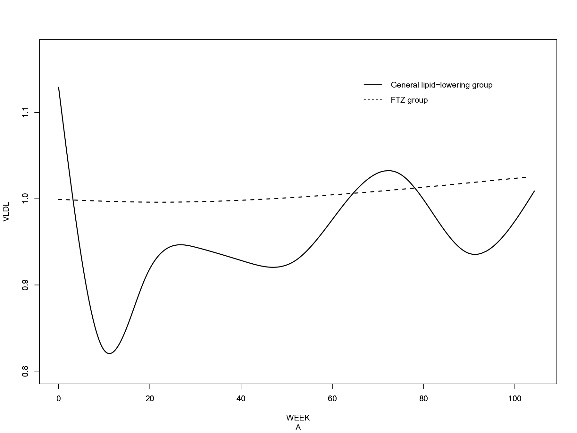

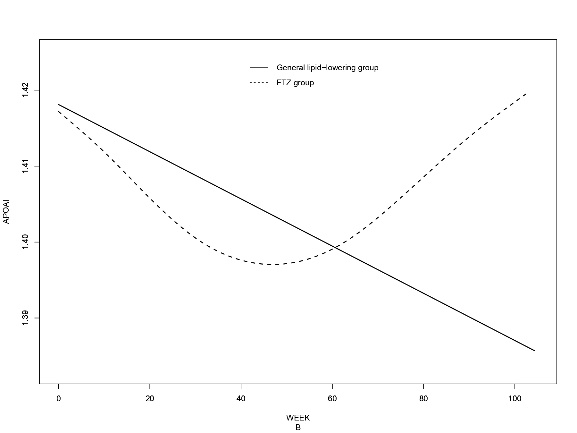

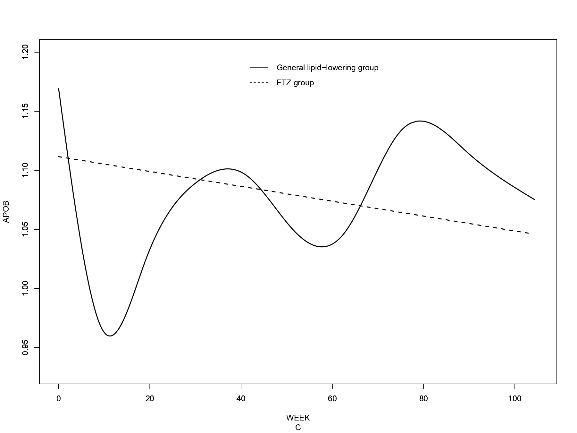

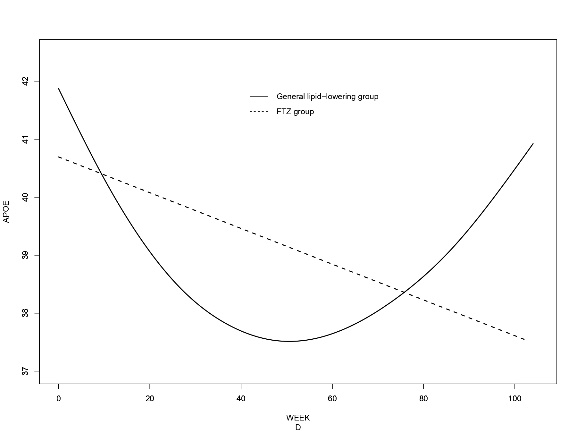

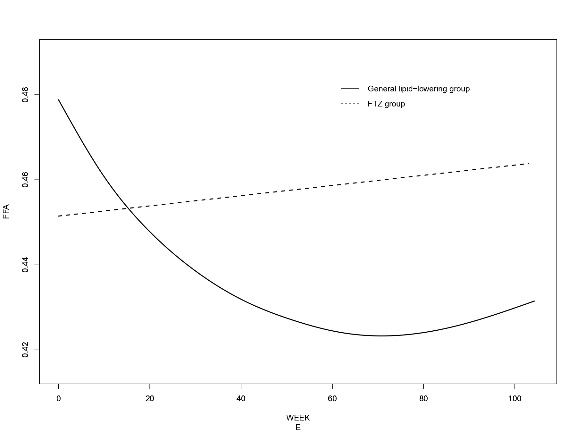


Figure S9. Other lipid profile trajectories two years after the initial treatment. Adjusted for age, sex and comorbidities.
